# Froin’s Syndrome: A Comprehensive Review of the Literature and the Addition of Two New Cases

**DOI:** 10.3390/neurolint16050083

**Published:** 2024-09-29

**Authors:** Lucas Jacobs, Bertil Delsaut, Marta Lamartine S. Monteiro, Audrey Cambier, Ibrahim Alcan, Evelyne Maillart, Maxime Taghavi

**Affiliations:** 1 Department of Internal Medicine, Brugmann University Hospital, Université libre de Bruxelles (ULB), 1020 Brussels, Belgium; 2 Department of Nephrology and Dialysis, Brugmann University Hospital, Université libre de Bruxelles (ULB), 1020 Brussels, Belgium; 3 Department of Neurology, University Hospital Tivoli, Université libre de Bruxelles (ULB), 7100 La Louvière, Belgium; 4 Department of Neurology, Brugmann University Hospital, Université libre de Bruxelles (ULB), 1020 Brussels, Belgium; 5 Department of Radiology, Brugmann University Hospital, Université libre de Bruxelles (ULB), 1020 Brussels, Belgium; 6 Department of Infectious Diseases, Brugmann University Hospital, Université libre de Bruxelles (ULB), 1020 Brussels, Belgium

**Keywords:** cerebrospinal fluid, Froin, Froin’s syndrome, paralysis, paresis, proteinorachia, spinal block, xanthochromia

## Abstract

Background. Froin’s syndrome (FS) is a rare entity with uncertain prevalence and prognosis, defined by a pathognomonic triad: cerebrospinal fluid (CSF) xanthochromia, elevated protein levels in the CSF, and hypercoagulated CSF, usually obtained through lumbar puncturing below the level of a partial or complete spinal block. Methods. We conducted a comprehensive review of the literature on FS from its first description in 1903 to December 2023, utilizing PubMed and Google Scholar, and included two new cases from our clinical practice. Results. We describe two patients who suffered from Froin’s syndrome secondary to spinal abscesses. According to our review, FS is caused by neoplasia in 33% of cases, non-malignant mechanical causes in 27%, infections in 27%, non-infectious inflammatory processes in 6%, and vascular in 6%. The most prevalent symptoms are paraplegia/paraparesis (64%), back pain (38%), altered mental state and/or confusion (23%), sciatica (17%), headaches (17%), leg sensory defects (17%), and urinary retention (14%), and are thought to be linked with the underlying causes rather than the CSF characteristics. FS holds a poor prognosis: only 22% recuperate fully after treatment, 22% die due to the cause leading to FS, and 14% retain sequelae. Conclusions. Xanthochromia and proteinorachia >500 mg/dL are not specific to any single pathological condition, but indicate defective CSF recirculation and spinal block, causing diffusive and/or inflammatory processes resulting in the hyperproteinosis and coagulation of the CSF. We reviewed the pathophysiology, etiologies, symptoms, outcomes, and workups of Froin’s syndrome according to the existing medical literature.

## 1. Introduction

Froin’s syndrome (FS) was first described in 1903 by Georges Froin [1] and remains to this date a rare, poorly known entity with uncertain prevalence and prognosis.

FS is defined by a pathognomonic triad: cerebrospinal fluid (CSF) xanthochromia, elevated protein levels in the CSF, and hypercoagulated CSF, usually obtained through lumbar puncturing below the level of a partial or complete block of the spinal canal [2]. In the first article attempting to apprehend the pathophysiology of FS in 1924, Joseph Godwin Greenfield described the following: “Among the many changes which the CSF may undergo, certainly the most striking to the clinical observer, is a yellow coloration, associated to the formation in the fluid, soon after it is received in a test-tube, of a coagulum, which may be so firm as to allow of the tube being turned upside down without a drop of fluid escaping” [2]. Since then, a few case reports on this syndrome have been published, referring to many different causes. In this article, we aim to present two original cases of FS, representing new additions to the very limited number of reported instances of Froin’s syndrome. By including these cases in our review, we expand the current knowledge base on this rare condition, offering further insights into its clinical presentation, diagnostic challenges, and outcomes. These cases contribute to the small but growing body of literature, reinforcing the importance of recognizing Froin’s syndrome in clinical practice.

## 2. Materials and Methods

**Figure 1:**
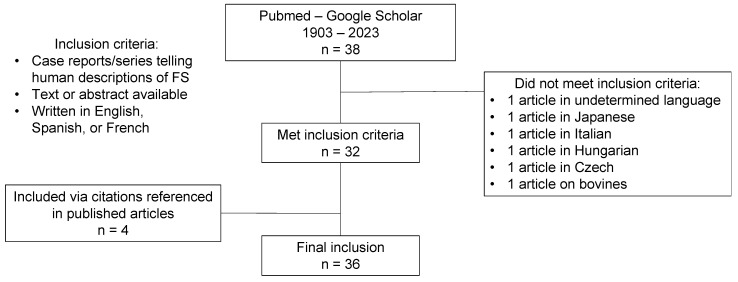
Literature screening process.

We performed a comprehensive literature review to identify clinical presentations as well as CSF findings, etiologies, treatments, and outcomes of Froin’s syndrome. The literature review includes all Froin’s syndrome patients. We used an electronic search of PubMed and Google Scholar databases for articles published between January 1903 and December 2023. The search terms were “froin syndrome” OR “froin’s syndrome” OR “pseudo-froin” OR “Nonne-Froin sign”. The references cited in published papers were also reviewed. We included case reports and case series featuring human descriptions of FS whose manuscripts or abstracts were available and written in English, Spanish, or French. Their titles and abstracts were reviewed first and, if relevant, the full text was obtained. We excluded a few non-English written articles whose abstracts or manuscripts were unobtainable for translation. Figure 1 presents the screening and selection of articles on Froin’s syndrome.

## 3. Two New Clinical Cases

### 3.1. Patient 1

A 67-year-old patient was admitted for Escherichia coli and Staphylococcus aureus septicemia secondary to cellulitis and osteitis in the setting of acute arterial ischemia and the recent amputation of his left toes. Upon admission he presented flaccid tetraplegia (force 1/6), absent myotatic reflexes, mute plantar responses, hypopallesthesia, and profound sensory defects in all limbs, dating back to 3 weeks before. He was hemodynamically stable and had no fever. His biology showed an elevated c-reactive protein and leucocytes (respectively, 148 mg/L and 15.000/µL). An electromyograph showed the absence of a distal motor response, as well as a severe hypovoltage of the proximal response in the muscles of the lower limbs, and a moderate hypovoltage of the motor response in the muscles of the upper limbs tested. Sensitive conduction was quasi-absent in the lower limbs and severely decreased in the upper limbs.

**Figure 2:**
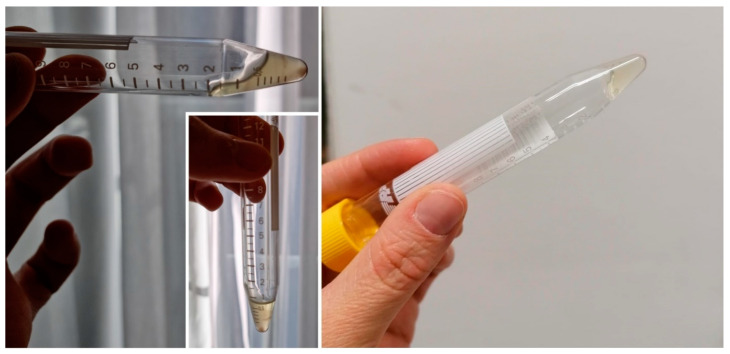
Xanthochromic coagulated aspect of CSF in patient 1.

**Figure 3:**
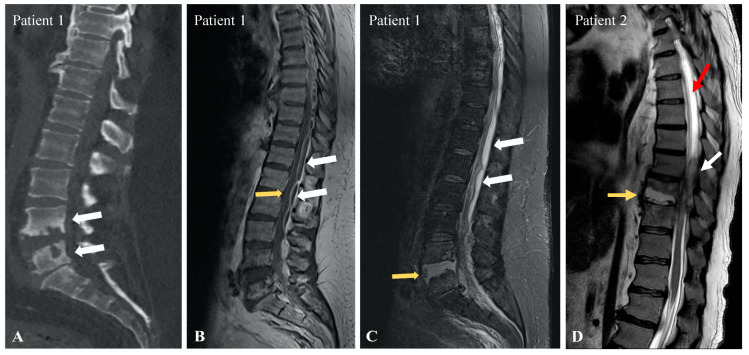
(**A**): Lumbar Computed Tomography showing L4 and L5 erosions (white arrows) due to L4-L5 spondylodiscitis. (**B**): Lumbar sagittal T1-weighted magnetic resonance imaging (MRI) showing multiple epidural abscesses (white arrows) with compressions of the spinal canal (yellow arrow). (**C**): Lumbar sagittal T2-weighted Short Inversion Time Inversion Recovery (STIR) MRI showing L4-L5 discitis (yellow arrow) with epidural abscesses (white arrows). (**D**): Thoraco-lumbar sagittal T2-weighted MRI showing T9–10 spondylodiscitis (yellow arrow), with epidural abscess (white arrow) and hypersignaling of the CSF above the compression (red arrow).

Lumbar puncture at the L3-L4 level revealed xanthochromic CSF, which coagulated within a few seconds (Figure 2). The analysis showed hyperproteinorachia of 12,610 mg/dL without hypoglycorachia. CSF bacterial cultures, viral PCR, and mycobacterium tuberculosis cultures were negative. Medullar magnetic resonance imaging (MRI) showed a severely narrowed spinal canal from C1 to C7 along with L4-L5 erosions due to spondylodiscitis (Figure 3A) and multiple spinal epidural abscesses, ranging from the T3 to L3 level with a mass effect on the spinal cord (Figure 3B). The MRI also showed L4-L5 discitis (Figure 3C). Decompression surgery and IV oxacillin associated with oral ciprofloxacin brought little immediate clinical benefit. The abscess cultures obtained from spinal surgery were positive for methicillin-sensitive Staphylococcus aureus. After surgery, our patient regained little sensitivity or motricity, however, becoming hemodynamically unstable two days after the surgery. He finally died of acute kidney injury and sepsis in the intensive care unit, one week after surgery.

### 3.2. Patient 2

A 56-year-old patient was admitted to the emergency department for lower back pain that had been evolving for several days. He was known to have alcoholic cirrhosis, with a lapse in follow-up for 3 years, complicated by portal hypertension and thrombocytopenia. The patient was obese and had type 2 diabetes and metabolic syndrome. He was chronically treated with metformin 850 mg, vidagliptin 50 mg, gliclazide 60 mg, and pantoprazole 40 mg, and continued to drink five glasses of wine per day.

Upon first examination, there was no neurological deficit or notable clinical abnormality. A lumbar CT scan revealed facet joint arthrosis at L4-L5-S1, and joint injections with corticosteroids were planned for the following week. A week later, in the physical medicine department, blood tests revealed significant inflammatory syndrome (C-reactive protein of 191 mg/L with neutrophilic leukocytosis), despite remaining afebrile. The patient was then transferred to the department of internal medicine. There, he would gradually deteriorate cognitively, becoming increasingly drowsy and disorientated, developing profound sensory deficits in all limbs along with flaccid tetraplegia, hypopallesthesia, and urinary retention.

In addition to blood cultures and urine cultures, an initial lumbar puncture would yield a dry tap. The next day, a second lumbar puncture would reveal drops of xanthochromic and coagulated material, where analyses were hindered by the coagulum. A new CT scan aimed at the lumbar region showed no causal lesion. A medullar MRI performed a few days later finally demonstrated a T9-T10 spondylodiscitis along with a right-posterior epidural abscess on T7, T8, and T9, causing significant compression of the spinal cord with hypersignal of the CSF in T2-weighted images above and below the level of compression (Figure 3D). Simultaneously, blood cultures were positive for Streptococcus constellatus, and aortic cusp endocarditis was found. The patient was treated with Penicillin G 4× 6,000,000 units, and surgery was performed to cure and drain the epidural abscess. The abscess cultures obtained from spinal surgery confirmed the presence of Streptococcus constellatus. Approximately 3 weeks post-procedure, the patient would be transferred to the intensive care unit (ICU) for septic shock and Escherichia coli septicemia secondary to a sacral bedsore. The patient developed acute anuric kidney failure, needing continuous extrarenal epuration, and decompensated cirrhosis with important ascites, moderate hyperbilirubinemia, moderate hyperammonemic encephalopathy, pleural effusions, and moderate coagulopathy. Twelve weeks after spinal decompression, there was no improvement in motor or sensitive function, despite physical therapy. The patient ultimately died from hepatic failure secondary to Escherichia coli septicemia.

## 4. Discussion

Froin’s syndrome was first described in 1903 by Georges Froin [1] and is defined by the association of hyperproteinorachia, xanthochromia, and hypercoagulated CSF (5). There are no data available regarding the incidence and the prevalence of FS. We found that 80% of FS cases described in the literature were in men, and the mean age was 51 years old.

That the CSF occasionally contained yellow pigment was discovered soon after lumbar puncture had been adopted as a routine clinical procedure [3]. This yellow discoloration of the CSF was observed by Millian and Chiray in 1902, in cases of subarachnoid hemorrhage, and they proposed the term “xanthochromia” to describe the phenomenon [4]. Georges Froin (1874–1964) in 1903 reported three clinical cases in which the CSF was not only yellow but also coagulated, and with an increased lymphocyte count. An insight into the mechanisms of xanthochromia was subsequently provided in 1936 by Robinson and Miller, who produced experimental compressions of the spinal cord in dogs and later recovered a xanthochromic high-protein-containing CSF [5].

### 4.1. Pathophysiology and Etiologies

**Figure 4:**
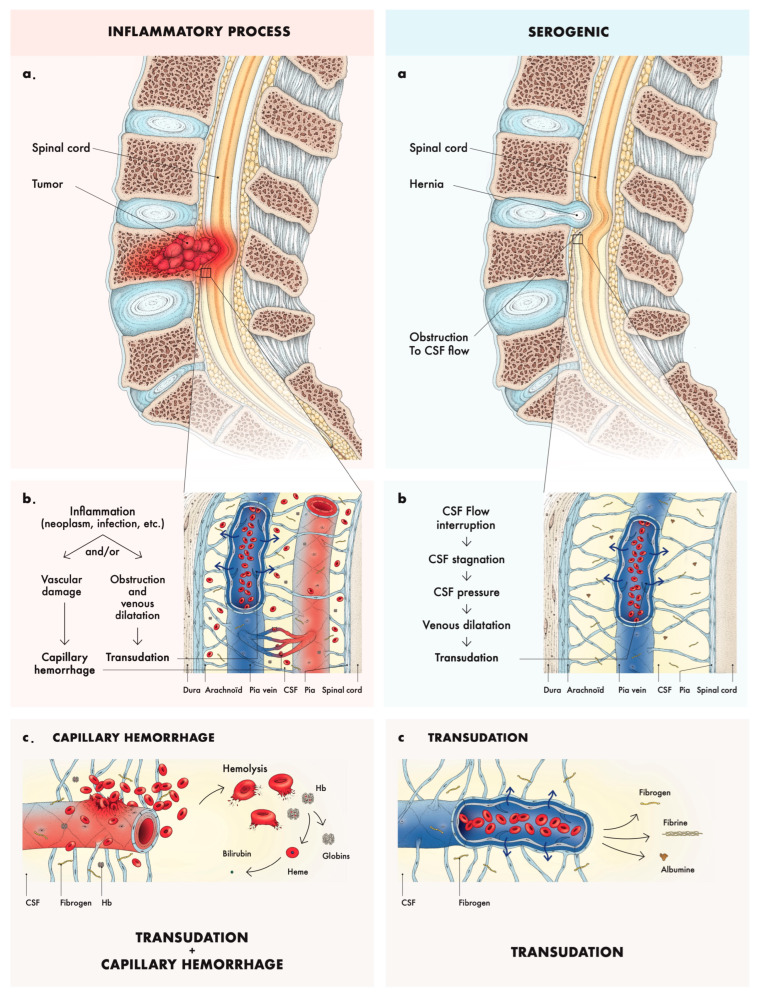
Pathophysiology of Froin’s syndrome. Legend: Froin’s syndrome can be divided into two pathophysiological mechanisms: inflammatory (**a.**) and serogenic (**a**). Inflammatory obstruction and process creates vascular damage and capillary hemorrhage (**b.**) and leaking of vascular content and hemolysis (**c.**). Serogenic process comes from pure mechanical obstruction of CSF flow (**a**) leading to transudation (**b**) and leaking of vascular content without hemolysis (**c**).

Xanthochromia (yellow-colored CSF) and proteinorachia >500 mg/dL are not pathognomonic of any one pathologic state, but are dependent on certain factors common to many. The pathophysiology of xanthochromia and proteinorachia can be divided into two different mechanisms (see Figure 4a): a serogenic origin (i.e., transudative processes) or an inflammatory/hemolytic origin (i.e., hemorrhage and hemoglobin transformation into bilirubin), both reflecting a breach of the blood–brain barrier.

The serogenic mechanism (sometimes referred to as pseudo-FS) is characterized by the absence of red blood cells or hemoglobin in the CSF, and is usually seen in cases of spinal cord compression with obstruction to the spinal canal. There is diminished pressure in the culdesac, as connection with the spinal fluid above the compression has been cut, and, at the site of compression the dilated pial veins induce transudation (Figure 4b). It includes cases of a herniated disk or neoplasm [6]. The flow of CSF in the perivascular space around near-surface pial arteries is pulsatile, driven primarily by oscillations of the artery wall due to the heartbeat, with a net flow in the direction of the blood flow [7]. With the force moving the CSF being proportional to pressure gradient multiplied by the area, the elasticity and compressibility of the dura could be susceptible to the caudo-cranial motion of CSF due to a wave pressure generated in the lumbar region, allowing a distention of the dura. This wave pressure would allow the CSF to progress passed the obstacle, while the less energetic rebound wave does not allow for the CSF to return as easily passed the compression. Perhaps the development of a low pressure below a partial block in FS may be due to the evacuation of the lumbar CSF space by muscular exertion [8]. The inflammatory/hemolytic mechanism is characterized by capillary hemorrhages in addition to exudation and transudation from a tumor itself, or from hematogenous factors, in loculated areas of the subarachnoid space, sequestered from cerebrospinal fluid circulation. It includes cases of inflammation, neoplasm, or trauma (Figure 4b). The color of the fluid is then also due to dissolved hemoglobin or its derivatives (Figure 4c).

Among all cases of Froin’s syndrome found in our literature review, the mean proteinorachia was 2800 mg/dL. Proteinorachia >500 mg/dL usually indicates a defective CSF recirculation and spinal block (i.e., spinal epidural abscess, tumors, degenerative stenosis, or herniations) [2,3,4,5], causing diffusive and/or inflammatory processes resulting in hyperproteinosis and hypercoagulation [9]. Hyperproteinorachia is aided by venous congestion below the level of compression [10] or by meningeal/spinal cord inflammation below an area of meningeal adhesion [9], and can worsen because of exudation from active tissue, whether malignant or infectious [10,11]. The CSF usually contains high levels of fibrin, fibrinogen, albumin, and often bilirubin. The hypercoagulated CSF may be explained by a soluble fibrin monomer complex [12] or the high fibrinogen level [6]. Although, whether increased protein levels can significantly affect CSF viscosity is a matter of debate; existing data suggest that it would not be significant [13]. Etiologies of FS are presented in Table 1.

### 4.2. Clinical Features

There is no description of specific symptoms of FS in the literature. The clinical manifestations of FS must be related to its cause rather than to any clinical effect of proteinorachia on its own. Indeed, it has been shown that, at body temperature, elevated proteinorachia do not significantly influence CSF viscosity [13]. In fact, some FS were incidentally found [39]. The mean proteinorachia of FS patients is around 2800 mg/dL.

However, since FS has a broad differential diagnosis, we have collected all descriptions of FS to provide readers with a range of suggestive symptoms. In the literature, the most common associated symptom was leg paralysis or paresis in 64% of cases; flaccid or spastic character was often not specified, with 4 cases being flaccid, 3 being spastic, and 13 unknowns. Back pain was experienced in 38% of patients, and altered mental state and/or confusion was experienced in 23% of cases. We further saw 17% with sciatica and headaches, 17% with sensory defects, and 14% with urinary retention. The symptoms of FS are presented in Table 2.

Hydrocephalus has been described in FS, which was probably secondary to inflammation and protein accumulation near the foramina of Luschka and Magendie at the base of the brain [35]. Finally, subependymal giant cell tumors (SGCTs), often occurring near the foramen of Monroi, can cause the frontal horn of the lateral ventricle to become an isolated “intracranial cul-de-sac”, mimicking the pathological conditions of Froin’s syndrome. This phenomenon of increased CSF coagulability is a unique feature of SGCTs, making it arguably an “intracranial variant” of the classic observation made in Froin’s syndrome [40].

### 4.3. Workup

Lumbar puncture is of certain importance for the diagnosis of FS. The latter can be difficult, as some cases, like that of our second patient, who reported a dry tap [11,32,36]. Indeed, Soon-Kul Kwon and Mi-Woon Kim reported cases of FS with low spinal pressure, below 1 cmH2O, and a thick density of the CSF, which was difficult to assess and did not drain freely upon lumbar puncture [25]. A normal CSF protein level ranges from 10 to 50 mg/dL [41]. The CSF analysis in FS shows a wide range of white blood cells (WBCs) and red blood cells (RBCs), probably related to the underlying etiology. Therefore, WBC and RBC counts are often not useful for FS diagnosis, but could point towards its underlying etiology.

Previous case series have described an increased signal intensity of CSF caudal to a level of spinal block when compared to the proximal level. This signal change on MRI has also been referred to as “pseudo-Froin” [12]. Interestingly, the signal intensity does not correlate with the protein content in in vitro studies [42]. One hypothesis would be that some macromolecules derived from cell membranes in association with paramagnetic species may contribute to the detectable signal changes seen in vivo [12]. Our first patient did not have an increased CSF T2 signal on MRI, while our second patient did have an increased signal in the CSF proximal and caudal to the obstruction; this observation has been shown to be inconsistent in the literature.

In case of xanthochromic CSF, a spectrophotometric analysis is recommended in order to differentiate a traumatic spinal tap and a true intracranial bleed [41]. Also, clinicians should perform routine CSF analysis (i.e., total protein levels, albumin, immunoglobulin, glucose, lactate, cell count, and cytology) immediately after collection [41,43].

### 4.4. Outcomes

Despite surgical decompression, our two patients would not regain their neurological functions until their deaths weeks later. The diagnosis delay likely contributed to the lack of recuperation. In the literature, FS holds a poor prognosis: only 22% recuperate fully after treatment, 22% die due to the cause leading to FS, and 14% retain sequelae (mostly severe), while 36% of articles did not specify the outcome. Table S1 provides a summary of all the cases of FS, describing the clinical presentation, the CSF findings, the treatment, and the outcome (see the Supplementary Materials).

## 5. Conclusions

Froin’s syndrome, though rare and likely underdiagnosed, is primarily documented through isolated case reports. This review provides an overview of the history, pathophysiology, etiologies, clinical manifestations, and outcomes of FS. Clinicians should remain vigilant of FS’s characteristic symptoms, imaging, and CSF findings to facilitate timely diagnosis and treatment, thereby improving prognosis. In cases without clear CNS pathology, FS should prompt investigation for a transudative or exudative cause in the spinal cord.

## Figures and Tables

**Table 1:** Etiologies of Froin’s syndrome in the literature.

Causes of Froin’s Syndrome	References
Neoplasia (33%)	
Spinal ependymoma	[11,12,14]
Multiple myeloma	[15]
Primary central nervous system tumors	[16,17,18]
Leptomeningeal involvement in hematological malignancy	[19]
Leptomeningeal carcinomatosis in malignant melanoma	[20]
Glioblastoma	[21]
Solid tumors metastasis	[22,23]
Mechanic (27%)	
Degenerative stenosis	[12,24]
Herniation	[12]
Trauma	[10,25]
Iatrogenic (post cerebral stereotactic needle biopsy)	[26]
Spinal cord injury	[27]
Spinal epidural lipomatosis	[28]
Dermoid tumor	[12]
Infectious (27%)	
Tuberculosis meningitis, Pott disease, tuberculoma of the conus medullaris	[29,30,31]
Bacterial abscess	[32,33,34]
Varicella-zoster virus encephalitis	[10,35]
Vascular (6.5%)	
Subarachnoid hemorrhage	[36]
Necrotizing vasculitis	[35]
Inflammatory (6.5%)	
Hypertrophic pachymeningitis	[37]
Neurosarcoïdosis	[38]

**Table 2:** Symptoms of Froin’s syndrome in the literature.

Symptoms of Froin’s Syndrome	Number of Cases (%)
Paralysis/Paresis	22 (64)
Back pain	13 (38)
Confusion/Altered mental state	8 (23)
Leg pain/Sciatica	6 (17)
Headache	6 (17)
Hypoesthesia/Anesthesia	6 (17)
Urinary retention/Incontinence	5 (14)
Visual loss	1 (3)

## Data Availability

No new data were created or analyzed in this study. Data sharing is not applicable to this article.

## Supplementary Materials

The following supporting information can be downloaded at https://www.mdpi.com/article/10.3390/neurolint16050083/s1: Table S1: Literature review of articles describing Froin’s syndrome in the literature.
