# Supplementary material for: Froin’s Syndrome: A Comprehensive Review of the Literature and the Addition of Two New Cases
Source: Neurol Int. 2024 Sep 29;16(5):1112–21. doi: 10.3390/neurolint16050083 (PMC11510248; doi:10.3390/neurolint16050083)
Supplement: Supplementary file 1 [file neurolint-16-00083-s001.zip › Table S1 - Froin.pdf]

Table S1. Literature review of articles describing Froin's syndrome in the literature

Year	Authors	Study type	Gender	Age	Presentation	Etiology	Aspect of CSF	CSF protein level [mg/dl]	Treatment	Outcome	Ref.
2024	Jacobs L, *et al*	Case report	M	67	Flaccid tetraplegia, hypopallesthesia, and hypoesthesia in all limbs.	Spinal stenosis from C1 to C7, epidural abscesses ranging from T3 to L3	Xanthochromia<br>Highly viscous<br>Coagulated	12610	Antibiotics, surgical decompression	Death related to kidney injury and sepsis.	
2024	Jacobs L, *et al*	Case report	M	56	Confusion. Progressive flaccid tetraplegia, hypoesthesia in all limbs. Urinary retention.	T9-T10 spondylodiscitis. Epidural abscess ranging from T7 to T9 causing important canal stenosis	Xanthochromia<br>Coagulated	NA	Antibiotics, surgical decompression	Death related to hepatic failure and sepsis.	
2023	Fan JL, *et al*	Case report	W	26	Back pain. Visual loss. Headache.	Myxopapillary ependymoma	Dry tap<br>Few CSF drops<br>Xanthochromia	NA	Surgical tumor removal	Deteriorating vision.	[11]
2023	Fries FL, *et al*	Case report	M	62	Past history of VP shunt placement. Worsening tetraparesis. Impoverishment of speech production.	Cervical canal stenosis. Subarachnoid hemorrhage & ventricular congestion.	Above VP shunt:<br>No color<br>IgG 60 mg/l<br>RBC normal<br>WBC normal<br>Below VP shunt:<br>Xanthochromia<br>IgG 1160 mg/l<br>RBC normal<br>WBC normal	Above VP shunt:<br>70<br>Below VP shunt:<br>930	Rehabilitation	Resumed previous neurological condition.	[33]
2022	Koc I, *et al*	Case report – Only abstract	F	48	Acute motor axonal neuropathy, absent deep tendon reflexes, hypotonia and paresthesia in the lower limbs. Headaches.	Leptomeningeal carcinomatosis	NA	NA	NA	NA	[27]

2022	Mantese CE, *et al*	Case report	F	64	Confusion, walking difficulty, lethargy.	Tuberculous myelitis with CSF block	Color and aspect not described Lymphocytic pleocytosis	590 – 2321	Steroids	Regained consciousness. Slow recuperation of paraparesis.	[39]
2022	Aguilar Jaldo MP, *et al*	Case report [Spanish]	M	79	Mechanical cervico-dorsal pain, progressive weakness of the lower limbs, and walking difficulty.	Epidural abscess	Xanthochromia Glucose 52 mg/dl RBC 479/µl WBC 10/µl	773	Antibiotics	No clinical sequelae.	[41]
2021	Nizam A, *et al*	Case report – Only abstract	M	38	NA	Leptomeningeal myelomatosis related to multiple myeloma	NA	NA	NA	NA	[24]
2021	Chotmongkol V, *et al*	Case report	M	78	Dull ache in both thighs, and progressive paraparesis	Tuberculoma of the conus medullaris	Xanthochromia Glucose 58 mg/dl RBC NA WBC 23/µl	2181	Isoniazid, rifampicin, pyrazinamide, ethambutol	Dull ache gradually disappeared, and motor power improved slightly.	[37]
2020	Grewal P, *et al*	Case report	F	60	Headache, subarachnoid hemorrhage with intraventricular hemorrhage and mild hydrocephalus.	Suspected formation of septi, spinal arachnoiditis following subarachnoid hemorrhage	Dry tap Thick viscous Xanthochromia Glucose 36 mg/dl RBC 238/µl WBC 12/µl	1428	External ventricular drain, coil embolization	Asymptomatic	[17]
2020	Sànchez Carteyron A, *et al*	Letter to the editor	M	Early 40s	Subacute encephalopathy, glioblastoma multiforme responsible for a temporal horn entrapment.	Post-biopsy transparenchymatous canal, connecting the stagnant temporal CSF to the subarachnoid space	Xanthochromia Extremely viscous Glucose 22 mg/dl RBC NA WBC NA	3000	NA	Death 18 days post-admission	[34]

2019	Garispe A, *et al*	Case report	M	55	Motor vehicle collision, 4 hours delayed mental status decline, pyrexia and HIV positivity [CD4 count of 8 cells/µl].	Traumatic C5-6 cervical stenosis with paravertebral fluid and edema, and varicella encephalitis superimposed	Xanthochromia<br>Glucose 100 mg/dl<br>RBC 78/µl<br>WBC 63/µl	1290	Acyclovir	Not evaluable	[10]
2019	Decramer T, *et al*	Case report	M	48	Severe spasticity after a C6 spinal cord injury due to motor vehicle accident 20 year earlier.	Spinal cord injury with intrathecal catheter obstruction	Xanthochromia, highly viscous.<br>Glucose 73 mg/dl<br>RBC few<br>WBC NA	3800	NA	NA	[35]
2019	Moscote-Salazar LR, *et al*	Letter to the editor	F	55	Posterior neck pain, progressive weakness in the upper and lower extremities, fever, adynamia.	Epidural C2-C5 abscess, positive for methicillin-sensitive Staphylococcus aureus	Color and aspect not described<br>Glucose 50 mg/dl<br>RBC NA<br>WBC 25/µl	1300	Cervical laminectomy, IV cloxacillin	Persistent severe paraparesis	[40]
2018	Maharjan K, *et al*	Case report	M	25	Paraparesis of both lower limbs, paraplegia with exaggerated lower limb deep tendon reflexes.<br>Tuberculous symptoms.	Tuberculous compressive epidural T6-9 abscess and discitis	Xanthochromia<br>Coagulated in 1 hour.<br>Glucose NA<br>RBC NA<br>WBC NA	1500	Isoniazid, rifampicin, pyrazinamide, ethambutol	Marked improvement in the paraplegia, though still dependent.	[38]
2018	Hale AT, *et al*	Case report	F	4	Severe nocturnal headaches, lower-back pain, bilateral leg pain.<br>Acute decompensation in neurological status overnight.<br>Incontinence.	Primary extradural intramedullary atypical teratoid/rhabdoid tumor	Xanthochromia<br>Viscous<br>Glucose 2 mg/dl RBC NA<br>WBC NA	1250	Dexamethasone	Multifocal strokes due to elevated ICP.<br>Death 18 Days after admission.	[25]
2018	Mirza S, *et al*	Case serie	M	66	Seven-year history of back pain.<br>Bilateral sciatica.	L3 Intradural, extramedullary ependymoma distal to the conus	NA	NA	Surgical excision of the tumor	Good postoperative recovery	[12]

2018	Mirza S, *et al*	Case serie	M	55	Three-months history of right sciatica. Reduced straight leg raise. Numbness in right L5 dermatome. Motor deficit of right foot dorsiflexion.	L3/L4 degenerative hypertrophic disc bulge producing severe central canal stenosis	NA	NA	Surgical decompression	Good postoperative recovery	[12]
2018	Mirza S, *et al*	Case serie	M	72	Longstanding history of back pain.	L2/L3 dermoid tumor	NA	NA	NA	NA	[12]
2017	Koton Y, *et al*	Case report	M	69	Ascending flaccid paraparesis. Back pain.	Intradural 25x11 mm metastasis of non-small cell lung carcinoma at T12-L1 level with extension into the extradural space	Xanthochromic Coagulated Viscous Glucose 21 mg/dl RBC/WBC 5/µl	2583	NA	NA	[32]
2017	Saga T, *et al*	Case serie – Only abstract [Japanese]	M	66	Consciousness disturbance.	Lymphomatous leptomeningitis	NA	NA	NA	NA	[28]
2017	Saga T, *et al*	Case serie – Only abstract [Japanese]	M	84	Bilateral leg pain.	Lymphomatous leptomeningitis	Cytology with lymphoma cells	NA	NA	NA	[28]
2016	Morren J, *et al*	Conference abstract	M	43	Four-week history of paraparesis, first flaccid, then spastic. Severe back and leg pain. Headache, fever, night sweats, weight loss. Urinary retention.	Neurosarcoïdosis with large obstructing subarachnoid granulomatous lesion	4 dry taps Neurosurgical intervention collecting viscous CSF	256	L4-L5 laminectomy	Death secondary to subarachnoid hemorrhage	[43]

2016	Dancel R, *et al*	Clinical image	M	64	Progressive paraplegia and hypoesthesia from L1-L2 levels	T11-T12 intradural, extramedullary schwannoma	Xanthochromia<br>Viscous<br>Glucose 45 mg/dl<br>RBC NA<br>WBC <1/µl	> 1500	Tumor resection. T11-12-L1 laminectomies	Improved sensations. No improvement in paraplegia.	[26]
2014	Gokahmetoglu, *et al*	Case report	M	72	NA	Large T12-L1 intramedullary tumor	Xanthochromia<br>Viscous<br>Spinal pressure <1cmH20<br>Glucose 46 mg/dl<br>RBC 0<br>WBC 50/µl	3115	NA	NA	[14]
2014	Ljevak J, *et al*	Clinical image	M	23	Disorientation, headache. Lower back pain. Nausea, vomiting, and fever.	Grade IV glioblastoma multiform	Unknown color<br>Coagulated	12050	NA	NA	[30]
2014	Ljevak J, *et al*	Clinical image	M	23	Mild paresis of right quadriceps with attenuated right patellar reflex.	Grade IV glioblastoma multiform	Xanthochromia<br>RBC 3<br>WBC 53/µl	17000	Chemotherapy and carmustine	NA	[30]
2014	Kwon SK, *et al*	Case report	M	54	20-year paraplegia	Burst spine trauma	Dry tap<br>Xanthochromia<br>Viscous<br>Coagulated<br>Glucose 46 mg/dl<br>RBC 0/µl<br>WBC 50/µl	3114	NA	NA	[19]
2013	Kimura S, *et al*	Case report	W	63	Headache, vomiting.	Dura mater metastasis of gastric cancer with subdural fluid [mucus] collection	Xanthochromia<br>Viscous<br>Coagulated<br>Glucose NA<br>RBC 0<br>WBC NA	NA	Burr-hole evacuation of the subdural collection	Coma after 2 weeks. Death due to disseminated intravascular coagulation.	[31]
2013	Mattsson, *et al*	Clinical image	W	60	Back pain. Incontinence	L1-L4 Ependymoma	Xanthochromia<br>Viscous<br>Coagulated<br>RBC 173/µl<br>WBC 2/µl	3100	Tumor resection	No sequellae	[23]

2012	Govindarajan R, *et al*	Letter to the editor	M	69	Paraplegia, mute plantar reflexes, lower back pain. Urinary retention.	Cervicothoracic epidural abscess Infarction of the thoracic cord	Initial dry tap Xanthochromia Glucose NA RBC 888/µl WBC normal	3295	Emergent open decompression and abscess drainage	NA	[18]
2010	Noake JR, *et al*	Abstract only	M	49	Paraparesis and sensory defects following respiratory tract infection.	Leptomeningeal carcinomatosis and cerebral metastasis of skin melanoma	NA	High protein levels	Steroids	Rapid deterioration, death	[29]
2000	Koch CA, *et al*	Case report	M	36	Back pain, muscle weakness [hip flexor muscles].	Spinal epidural lipomatosis	NA	620	Inhibitors of steroidogenesis	Good recovery	[36]
1998	Kleinschmidt-DeMasters BK, *et al*	Case report	M	54	Confusion, headache.	Multiple subpial hemorrhages and necrosis due to varicella zoster virus vasculitis	Color NA Aspect NA Glucose 100 mg/dl RBC 4430/µl WBC 1300/µl	1877	Intravenous acyclovir and foscarnet	Progressive worsening of neurological status. Coma. Death	[15]
1928	Wiersma D	Case report	M	22	Ascending flaccid tetraplegia and hypoesthesia. Back pain. Hyperesthesia and hyperalgesia above level of diminished sensations. Incontinence.	Pachymeningitis hypertrophica	Xanthochromia Aspect NA No RBC No WBC	NA	NA	Progressive worsening of neurological status. Death.	[42]
